# Plasma-Engineered N-CoO*_x_* Nanowire Array as a Bifunctional Electrode for Supercapacitor and Electrocatalysis

**DOI:** 10.3390/nano12172984

**Published:** 2022-08-29

**Authors:** Qi Wang, Tongtong Zhong, Zhou Wang

**Affiliations:** Key Laboratory of Liquid-Solid Structural Evolution and Processing of Materials of Ministry of Education, School of Materials Science and Engineering, Shandong University, Jinan 250061, China

**Keywords:** plasma, nitrogen doping, Co_3_O_4_, supercapacitor, electrocatalysis

## Abstract

Surface engineering has achieved great success in enhancing the electrochemical activity of Co_3_O_4_. However, the previously reported methods always involve high-temperature calcination processes which are prone to induce agglomeration of the nanostructure, leading to the attenuation of performance. In this work, Co_3_O_4_ nanowires were successfully modified by a low-temperature NH_3_/Ar plasma treatment, which simultaneously generated a porous structure and efficient nitrogen doping with no agglomeration. The modified N-CoO*_x_* electrode exhibited remarkable performance due to the synergistic effect of the porous structure and nitrogen doping, which provided additional active sites for faradic transitions and improved charge transfer characteristics. The electrode achieved excellent supercapacitive performance with a maximum specific capacitance of 2862 mF/cm^2^ and superior cycling retention. Furthermore, the assembled asymmetric supercapacitor (N-CoO*_x_*//AC) device exhibited an extended potential window of 1.5 V, a maximum specific energy of 80.5 Wh/kg, and a maximum specific power of 25.4 kW/kg with 91% capacity retention after 5000 charge–discharge cycles. Moreover, boosted hydrogen evolution reaction performance was also confirmed by the low overpotential (126 mV) and long-term stability. This work enlightens prospective research on the plasma-enhanced surface engineering strategies.

## 1. Introduction

At present, the prosperity and development of human society rely on the excessive consumption of fossil fuels, which inevitably causes severe energy crises and environmental pollution [1,2,3]. Optimizing energy structure through replacing conventional energy with clean renewable energy sources, such as solar, wind and tidal energy, has proven effective in releasing the contradiction [4,5,6]. The majority of renewable energy sources, however, come from natural forces which are highly reliant on the local climate, timing and geography. As a result, the regeneration and supply of renewable energy is discontinuous in both time and space, making energy storage equipment crucial in the large-scale renewable energy applications [7,8]. Supercapacitors have received extensive attention due to their extremely high-power density, long cycle life and fast charge/discharge characteristics [9,10,11]. However, low-energy density still limits their practical application. In contrast, electrocatalytic water splitting is believed to be a promising strategy for H_2_ production, which can be fueled in the fuel cells and subsequently generate power with no pollutant emissions. Unfortunately, the efficiency of electrocatalysis is always restricted by the higher overpotential and slow kinetics in catalytic reactions [12]. Above all, there is an urgent need to develop high-performance supercapacitor and electrocatalytic devices, but this is still a significant challenge.

Since energy storage and electrocatalysis depend on the heterogeneous reactions that occur on the surface of active materials, surface engineering, including doping [13,14], morphology modification [15,16], preferential facet growth [17,18], defect engineering [19,20], and heterojunction construction [21,22], has been applied as a popular strategy to improve the performance. Among various strategies, morphology modification can directly affect catalytic activity via surface structure regulation, which varies in terms of active reaction area, ion transport and surface energy. Porous structure with abundant active reaction sites and favorable ion transfer characteristics are commonly believed favorable to electrode materials for supercapacitors and electrocatalysis [23,24]. Moreover, doping is another potent strategy, which can efficiently improve the activity by regulating the electronic structure and the chemical bonding conditions. Among diverse doping elements, nitrogen doping, one popular subject in both theoretical and experimental research, has been corroborated beneficial to transition metal oxides in the following three aspects: (1) the incorporation of nitrogen can improve the electron transport ability and reduce the Schottky barrier between the catalyst and electrolyte, thus lowering the overpotential required for water splitting [25]; (2) the injected N element can adjust the adsorption/desorption energy of the reaction intermediates via regulating the electronic structure, hence improving the reaction kinetics [26]; (3) the incorporated N element with lower electronegativity increases the proportion of covalent bonds, which improves the environmental tolerance, chemical stability and structural stability, thereby enhancing the resistance to corrosion failure induced by acidic or alkali solutions [27].

The rare-earth oxide Co_3_O_4_, owing to its high theoretical capacity, controllable morphology and multiple valence states, has been considered as a competitive material in various fields, including energy storage, catalysis and sensing [28,29,30]. However, the poor conductivity and low cycling stability restrict its application as a high-performance active electrode material in supercapacitor and electrocatalysis. Previous studies have shown that constructing porous structure and introducing nitrogen doping can significantly improve the electrochemical performance of Co_3_O_4_. However, the reported modification approaches always involve high-temperature calcination processes [31,32], which are prone to induce agglomeration in the pristine nanostructure and negatively impact the activity of Co_3_O_4_.

In this work, the NH_3_/Ar plasma treatment was applied to conduct surface engineering on the hydrothermally prepared Co_3_O_4_ nanowires. Taking advantage of the energetic and activated plasma, porous Co_3_O_4_ nanowires with efficient nitrogen doping were obtained at a low temperature. In order to verify the synergistic effect of etching effect and nitrogen doping, systematic characterizations, including morphology, phase, electron structure, supercapacitive behavior and hydrogen evolution reaction (HER) performance, were performed on Co_3_O_4_ nanowires before/after NH_3_/Ar or pure Ar plasma treatment. Furthermore, the mechanism of the superior performance is discussed. Our work provides a new idea to developing high-activity materials and might enlighten the perspective research on the synergistic effect of different surface engineering strategies.

## 2. Materials and Methods

**Scheme 1:**
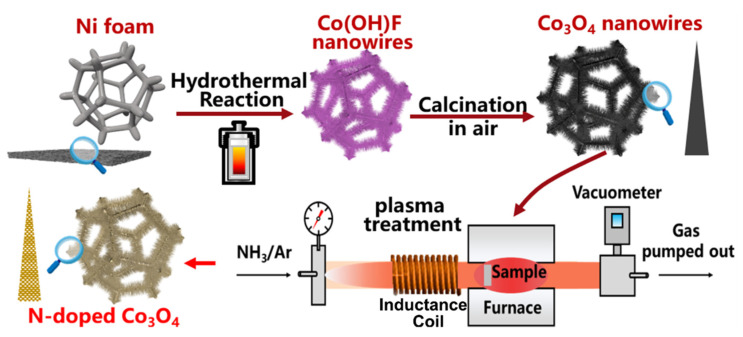
Schematic diagram of the fabrication procedures.

The fabrication procedures, including hydrothermal growth of Co_3_O_4_ nanowires and the subsequent low temperature (LT) plasma treatment, are outlined in Scheme 1.

### 2.1. Direct Growth of Co_3_O_4_ Nanowires on Ni Foam

Before the deposition, the Ni foam substrate (1 cm × 2 cm) was immerged in HCl (1 M) solution for 10 min to remove the oxide layer, then cleaned and ultrasonicated with acetone, ethanol, and deionized (DI) water for 15 min each. 

Firstly, the precursor Co(OH)F nanowires were prepared on Ni foam via a facile hydrothermal method [33]. Typically, 1.237 g of Co(NO_3_)_2_·6H_2_O, 0.315 g of NH_4_F, and 1.276 g of urea were dissolved in 59.5 mL distilled water. The whole mixture was stirred into a pellucid and uniform solution at room temperature. The obtained solution together with the dried Ni foam were transferred to a 100 mL Teflon-lined stainless-steel autoclave and heated in an electric oven at 120 °C for 5 h. After naturally cooling down to room temperature, the Ni foam was thoroughly washed with DI water under ultrasonication and dried at 70 °C for 6 h. Subsequently, the Co(OH)F nanowires were annealed in air at 350 °C for 2 h to obtain Co_3_O_4_ nanowires. The proposed synthesis reactions can be described as:

CO(NH_2_)_2_ + 3H_2_O = 2NH_4_^+^ + 2OH^−^ + CO_2_↑ (1)

Co^2+^ + F^−^ + OH^−^ = Co(OH)F↓ (2)

3Co(OH)F + 1/2O_2_ = Co_3_O_4_ + 3HF↑ (3)

### 2.2. Plasma-Induced Fabrication of N-Doped Co_3_O_4_

The N-doped Co_3_O_4_ nanowires were obtained by exposing the samples to NH_3_/Ar (10% NH_3_) plasma (13.56 MHz radio frequency source). First, the Co_3_O_4_ nanowires-loaded Ni foam was set vertically in the quartz tube chamber, and then the reactor was vacuumed down to 1 × 10^−5^ Pa based pressure followed by flowing NH_3_/Ar gas to working pressure of 40 Pa. The LT plasma process was controlled by regulating the plasma power and processing time. The optimized working condition was at a power of 300 W and a pressure of 40 Pa for 6 min under NH_3_/Ar plasma. Herein, the Co_3_O_4_ nanowires treated at a power of 300 W with varied operation time were denoted as N/Ar-t (t = 2, 4, 6, 8 and 10 min).

As control experiments, Co_3_O_4_ nanowires treated under high-purity Ar plasma at 300 W for 6 min were denoted as Ar-6.

### 2.3. Characterization

The scanning electron microscopy (SEM) was conducted on a JEOL 7800F ultra high-resolution field emission scanning electron microscope. The crystallography of the samples was determined by X-ray diffraction (XRD) which used a Rigaku D/Max-KA diffractometer with Cu Kα radiation (λ= 0.15418 nm). X-ray photoelectron spectra (XPS) measured on an Axis Supra (Kratos) using Mg Kα radiation of 1253.6 eV was to explore the chemical composition and valence of the surfaces.

### 2.4. Electrochemical Measurements

All electrochemical measurements were performed on the IVIUM Vertax electrochemical workstation. A series of electrochemical performance tests included cyclic voltammetry (CV), linear sweep voltammetry (LSV), galvanostatic charge-discharge (GCD), electrochemical impedance spectroscopy (EIS), electrochemical active surface areas (EASA), etc.

#### 2.4.1. Electrochemical Evaluation of Supercapacitor Performance

The supercapacitor performance was studied by forming a conventional half-test cell containing a three-electrode system comprised with an as-prepared sample as the working electrode, platinum (Pt) as the counter electrode and the Hg/HGO as the reference electrode, in 6.0 M KOH aqueous electrolyte.

The electrochemical performance of the assembled asymmetric supercapacitor (ASC) was measured in a two-electrode system. The N-doped Co_3_O_4_ electrode with an average mass loading of ~2 mg/cm^2^ worked as the cathode, while the functionalized activated carbon was used as the anode, the preparation of which was as follows. First, the commercial activated carbon was heated in 40% nitric acid at 60 °C for 3 h, and after centrifugal washing and drying, the obtained black powder was collected and named as AC-40. Then the as-prepared AC-40, carbon black and Polyvinylidene Fluoride (PVDF) at a mass ratio of 8:1:1 in N-methyl-2-pyrrolidone (NMP)were fully mixed and ground in an agate mortar. Finally, the obtained uniform slurry was evenly coated on the washed nickel foam, and then placed in a blast oven at 60 °C for 24 h.

In order to obtain the optimum device performance, the cathode and anode should achieve charge balance (q^+^ = q^−^). Therefore, the mass loading of AC-40 was calculated to be 4.74 mg using the following equation [34]:

(4) $$\frac{m_{+}}{m_{-}}=\frac{C_{s\text{–}}{\;\Delta V}_{-}}{C_{s+}{\;\Delta V}_{+}}$$

where *m* is the mass (g), *C_s_* is the specific capacitance (F/g) and Δ*V* is the potential window (V).

#### 2.4.2. Electrochemical Performance of Electrocatalytic Hydrogen Evolution

The HER of as N-doped Co_3_O_4_ electrode was also measured in the same three-electrode system as above with 1.0 M KOH (pH = 14) solution as the electrolyte. The potential measured against the Hg/HgO electrode was converted to the potential versus the reversible hydrogen electrode (RHE) according to E_RHE_ = E_Hg/HgO_ + 0.0591 × pH + 0.098. Before the measurements, the working electrode was activated in supporting the electrolyte for 50 cycles of CV between −0.8 V and −1.5 V at scan rate of 50 mV s^−1^. LSV measurements were performed in the potential range of −0.8 V–−1.5 V at the scan rate of 2 mV s^−1^.

### 2.5. Calculations

The areal capacitances (*Cs*, mF/cm^2^) of the electrodes and assembled ASC devices were calculated from GCD curves according to the following equation [35]:

(5) $$C_{s}=\frac{I\;\times \;\Delta t}{\Delta V\;\times \;s}$$

where *I*, Δ*t*, Δ*V* and *s,* refer to the constant discharge current (mA), discharge time (s), potential window (V) and the specific area (cm^2^), respectively.

The specific energy density (*E*, Wh/kg) and specific power density (*P*, W/kg) of the ASC devices were calculated from the discharge time obtained from the GCD plot using the following formulae [35]:

(6) $$E=\frac{I\;\int^{\;}V\;\mathrm{dt}}{m}$$

(7) $$P=\frac{E}{t}$$

where *I* is the discharge current (A), ∫*V* d*t* is the area under the discharge curve (Vh), *m* is the total mass of the active material (kg) and *t* is the discharge time (h) of the ASC device.

## 3. Results and Discussion

### 3.1. Structural Characterization

**Figure 1:**
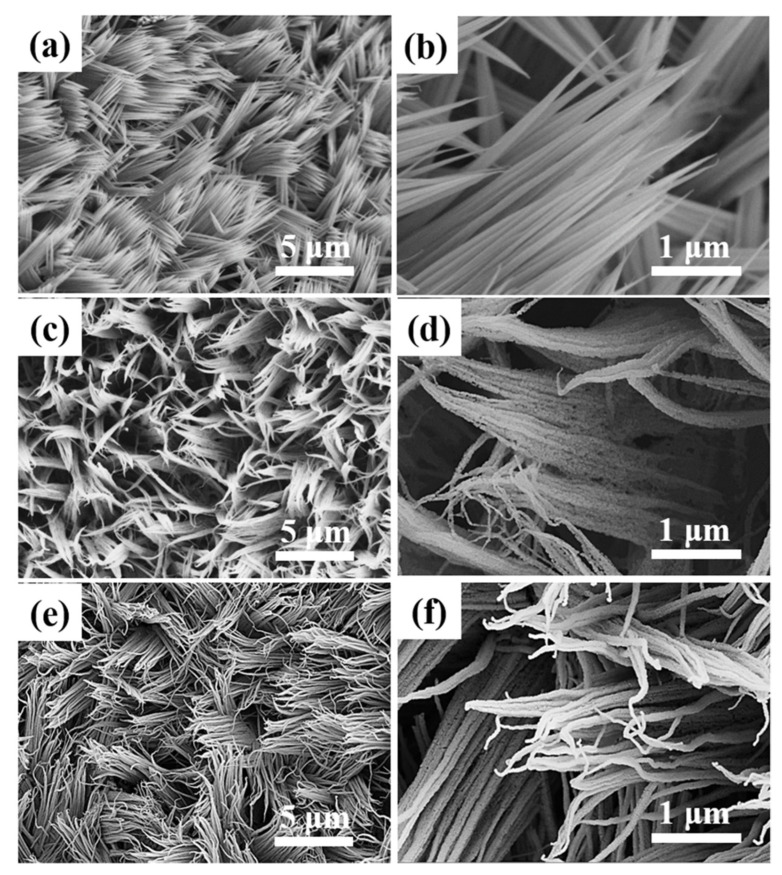
SEM images of (**a**,**b**) Co_3_O_4_, (**c**,**d**) N/Ar-6 and (**e**,**f**) Ar-6.

Hydrothermally prepared Co_3_O_4_ nanowires were controllably treated by NH_3_/Ar plasma with varied operation time to yield the N-doped Co_3_O_4_ nanowires (N/Ar-t). The morphologies of the obtained N/Ar-t were investigated by SEM imaging, taking Co_3_O_4_ and pure Ar-plasma treated Co_3_O_4_ nanowires as a comparison (Figure 1 and Figure S1). The pristine Co_3_O_4_ nanowires exhibit a typical nano-needle morphology with a compact and smooth surface (Figure 1a,b). It is obvious that the periodic surface structure was disrupted by the plasma treatment, whether it was conducted in NH_3_/Ar or a pure Ar atmosphere (Figure 1c–f). As shown in Figure S1a–d, the nanowires treated in NH_3_/Ar plasma for a relatively short time (2 min and 4 min) still maintained a compact surface structure. However, the originally straight needles gradually twisted from the tip and the surface turned rough. When the operation time was prolonged to 6 min, a rough porous structure with no obvious agglomeration and crack was obtained, which can be credited to the moderate treating time at a low temperature (Figure 1c,d). Further increasing of operation time induced gradual destruction to the porous nanowire structure, as can be seen from Figure S1e–h. When the operation time was extended to 8 min, the uniform nanowire array was destroyed with parts of nanowires assembled together and the other parts fractured. The longer operation time (10 min) caused severe damage to the nanowires, most of which were etched into short rods or even nanoparticles. As a control group, the morphology of Ar-6 was also investigated, which manifested identical surface features with N/Ar-6 (Figure 1e,f). The EDS-Mapping method was applied to make an approximate estimate of the doped N element (Figure S2) and the calculated concentrations of Co, O and N are summarized in Table S1, which verifies that increasing operation time can efficiently enhance the doping concentration of N in Co_3_O_4_. The N content reached 1.3 at% in the optimized N/Ar-6 sample.

**Figure 2:**
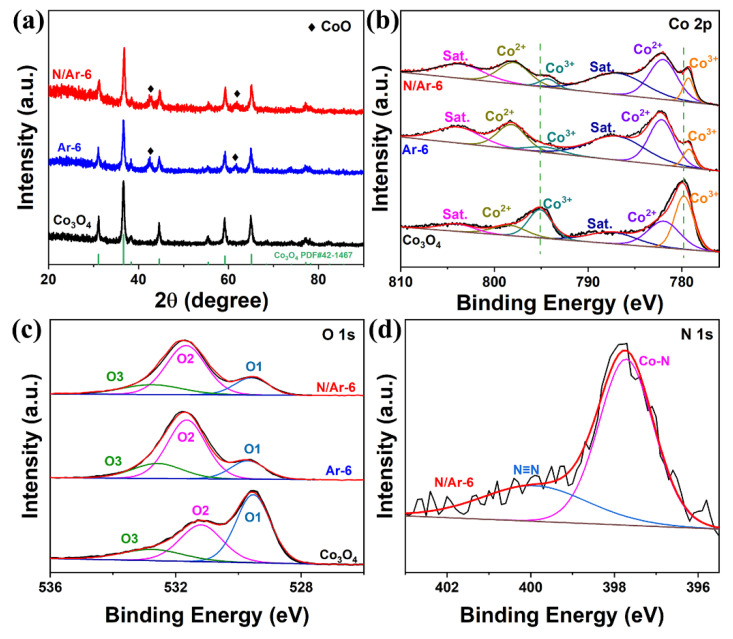
(**a**) XRD patterns, (**b**) Co 2p and (**c**) O 1s XPS spectra of Co_3_O_4_, Ar-6 and N/Ar-6. (**d**) N 1s XPS spectrum of HRTEM images of N/Ar-6.

The crystal phase of Co_3_O_4_, Ar-6 and N/Ar-6 was further confirmed by X-ray diffraction, as illustrated in Figure 2a. The diffraction pattern for pristine sample exhibited five strong peaks at 31.18°, 36.66°, 44.54°, 59.16° and 65.90°, which can be well-indexed to (220), (311), (400), (511) and (440) of Co_3_O_4_ (JCPDS No. 42-1467), respectively. No impurity peak was observed, validating the high purity of the obtained Co_3_O_4_. After plasma treatment, the XRD patterns of Ar-6 and N/Ar-6 were still mainly composed of the characteristic peaks of Co_3_O_4_, however two obvious signal peaks (marked by ♦) were recorded at around 42.5° and 61.5°, which can be indexed to the (200) and (220) of CoO (JCPDS No. 43-1004). The newly emerged peaks can be attributed to the reduction process during the plasma etching process. The identical patterns of Ar-6 and N/Ar-6 proves their consistent composition, and the absence of other peaks evinces that no cobalt nitrides were generated.

To examine the composition and chemical valences of Co_3_O_4_, Ar-6 and N/Ar-6, the XPS experiments were implemented correspondingly. The XPS survey spectra (Figure S3) confirm the coexistence of Co, O and C elements in all samples, while a weak signal peak of N element can be observed in the spectrum of N/Ar-6, suggesting the efficient nitrogen doping via NH_3_/Ar plasma. The high-resolution Co 2p spectra of all samples mainly feature two contributions (2p_1/2_ and 2p_3/2_). The 2p_1/2_ and 2p_3/2_ peaks of pristine Co_3_O_4_ are located at 795.12 eV and 779.76 eV, respectively (Figure 2b) [31,36]. However, the 2p_1/2_ and 2p_3/2_ peaks in the Co 2p spectra of Ar-6 and N/Ar-6 present an obvious shift towards high binding energy (BE). In order to get a more accurate assessment, each Co 2p spectrum was further decomposed into six peaks (two Co^3+^, Co^2+^ and satellite peaks), which demonstrate that the peak shift of 2p_1/2_ and 2p_3/2_ is due to the different Co^3+^/Co^2+^ ratio on the surface layer. The Co^3+^ accounts for a substantial part in Co_3_O_4_, while Ar-6 and N/Ar-6 are mainly made up of Co^2+^. The higher concentration of Co^2+^ can be ascribed to the preferred etching effect on lighter O element, which is in consistent with the detection of CoO by XRD analysis. O 1s core level spectra of all samples can be fitted into three distinct peaks at 529.53, 531.19 and 532.75 eV (Figure 2c), which are attributed to lattice oxygen (O1), low-coordination oxygen species (O2) and the adsorbed oxygen (O3), respectively [36]. According to the previous reports [16], the concentration of oxygen vacancies can be evaluated by the ratio of O2 peak. Apparently, Ar-6 and N/Ar-6 display much higher peak intensity of O2 than the pristine Co_3_O_4_, unraveling more abundant oxygen vacancies in both plasma-treated samples. Moreover, the doped nitrogen element in N/Ar-6 was investigated through N 1s core level spectrum (Figure 2d). Although low in intensity, the N 1s peak is readable and can be divided into two signals centered at 397.7 and 399.9 eV, which are originated from the Co-N bond and adsorbed nitrogen molecules (N≡N) [32]. The existence of Co-N signal verifies the efficient nitrogen injection into Co_3_O_4_ lattice.

### 3.2. Half Cell Studies

The conventional electrochemical tests based on half cells were operated to evaluate the supercapacitive performance. Previous to the systematic performance evaluation, a group of samples treated in NH_3_/Ar for different operation time were tested to obtain the optimum N/Ar-t electrode. As shown in Figure S4a, the CV curves for all the electrodes depict an asymmetric shape with a distinguishable redox peak signal, illustrating their pseudocapacitive energy-storage mechanism. At first, the area of the CV loop, representing the capacitance, increased with the prolonging operation time. The area of CV curve reached the largest value at the operation time of 6 min while a further increase in the operation time led to a dramatically shrinking curve. The result can be explained by the evolution of morphology revealed by previous SEM tests. During plasma treatment, the generated energetic particles impact the Co_3_O_4_ nanowires and gradually etch the surface layer, producing a rough and porous structure. Meanwhile, the nanowires are well maintained due to the moderate etching degree. Therefore, the active area and the concentration of active reaction sites increase at the early stage, expressing an elevated trend in capacitance. However, the continuous etching eventually destroys the structure of nanowires and induces aggregation and fracture (as shown in Figure S1e–h), which finally results in decreased performance. The recorded GCD curves of N/Ar-t electrodes show an identical rule with CV curves (Figure S4b), apparently granting N/Ar-6 the optimum NH_3_/Ar plasma treated electrode.

**Figure 3:**
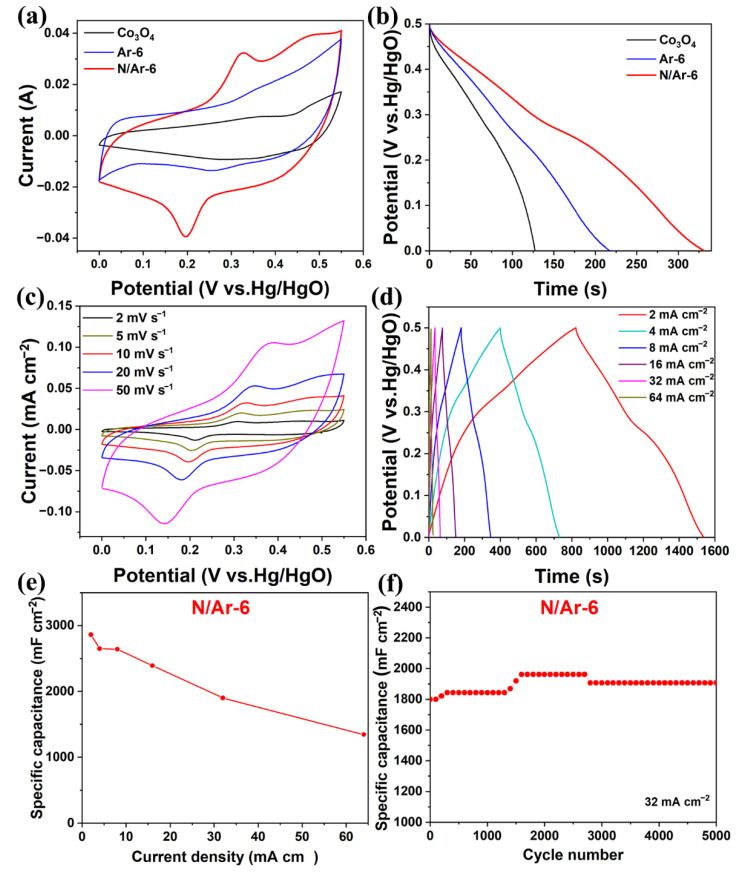
(**a**) CV curves and (**b**) GCD curves of Co_3_O_4_, Ar-6 and N/Ar-6. Supercapacitive performance of N/Ar-6: (**c**) CV curves under different scan rates (2–50 mV/s), (**d**) GCD curves under different current densities (2–64 mA/cm^2^), (**e**) Specific capacitance calculated from GCD curves in Figure 3d, (**f**) long-term cycling performance.

In order to scrutinize the effect of NH_3_/Ar plasma treatment, the supercapacitive performance of Co_3_O_4_, Ar-6 and N/Ar-6 was recorded for comparison. CV curves in Figure 3a deliver two noteworthy points: First, the increased capacitance of Ar-6 electrode indicates that the performance can be enhanced simply by etching effect, which expands the surface area and produces abundant active sites; secondly, the largest capacitance achieved by N/Ar-6 demonstrates the important role of nitrogen doping, which is believed helpful in enhancing the reactivity and facilitating electron transfer. Another interesting finding is the clear redox peaks on the CV curve of N/Ar-6, which is a sign of improved reactivity and better transfer behavior. Identical conclusions can also be drawn based on the study of GCD curves (Figure 3b).

The CV tests under different scan rates and GCD tests under diverse current densities were achieved on the optimized N/Ar-6 electrode to inspect the rate capability. Figure 3c depicts the CV curves recorded under the scan rate rang of 2 ~ 50 mV/s. Under a scan rate of 2 mV/s, two groups of redox peaks can be distinguished from the CV curve, which originates from the redox reactions between Co^2+^/Co^3+^ and Co^3+^/Co^4+^. The relative reactions are represented below [30]:

CoO + OH^−^ ↔ CoOOH + e^−^ (8)

Co_3_O_4_ + OH^−^ +H_2_O ↔ 3CoOOH + 3e^−^ (9)

CoOOH + OH^−^ ↔ CoO_2_ + H_2_O + e^−^ (10)

The specific capacitances of N/Ar-6 electrode under diverse current densities were calculated from the GCD curves (Figure 3d) and the obtained values are collected in Figure 3e. The specific capacitance reaches 2862 mF/cm^2^ at a low scan rate of 2 mA/cm^2^ and maintains 46.96% of the capacitance (1344 mF/cm^2^) at 64 mA/cm^2^, exceeding those of some previously reported nickel foam-based Co_3_O_4_ electrodes, such as ZnO/Co_3_O_4_ (1135 mF/cm^2^ at 1 mA/cm^2^) [37], Co_3_O_4_ (814 mF/cm^2^ at 1 mA/cm^2^) [37], Co_3_O_4_/NiO (2352 mF/cm^2^ at 1 mA/cm^2^) [38], Co_3_O_4_/Mn_2_O_3_ (1762 mF/cm^2^ at 1 mA/cm^2^) [38], Co_3_O_4_ (680 mF/cm^2^ at 1 mA/cm^2^) [38], ZnO@Co_3_O_4_ (1720 mF/cm^2^ at 2 mA/cm^2^) [39], Co_3_O_4_ (956 mF/cm^2^ at 2 mA/cm^2^) [39], nitrogen-doped carbon dots/NiO/Co_3_O_4_ (1183 mF/cm^2^ at 2/3 mA/cm^2^) [40]. The long-term cycling performance of the N/Ar-6 electrode was investigated up to 5000 cycles using GCD measurements at a current density of 32 mA/cm^2^. As shown in Figure 3f, the specific capacitance of the N/Ar-6 electrode manifested no trace of decay after 5000 cycles, illustrating its excellent long-term cycling performance. The increased specific capacitance at about 2000th cycle can be attributed to the activation of electrode during the cycling.

### 3.3. Asymmetric Supercapacitor Devices Performance

**Figure 4:**
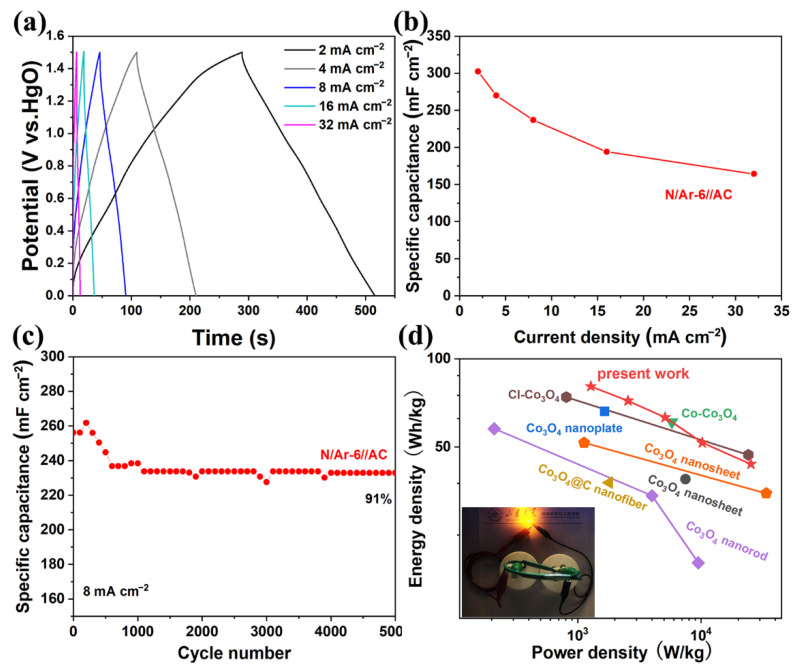
Supercapacitive performance of N/Ar-6//AC-40 asymmetric device: (**a**) GCD curves under different current densities (2–32 mA/cm^2^), (**b**) Specific capacitance calculated from GCD curves in Figure 4a, (**c**) long-term cycling performance. (**d**) Ragone plots of N/Ar-6//AC device and the recently reported asymmetric supercapacitor devices based on Co_3_O_4_//carbon. The insert shows LED powered by two N/Ar-6//AC devices in series.

The N/Ar-6//AC-40 asymmetric supercapacitor laboratory device was assembled to appraise the practicability of N/Ar-6 in real applications. The electrochemical performance of AC-40 half cell was presented in Figure S5. The supercapacitive performance of the asymmetric device was evaluated using CV and GCD measurements. Owing to the well-fitted cooperation of two electrodes, the device can steadily work under a potential window as wide as 0–1.5 V (Figure S6). Even under the low scan rate (2 mV/s), the CV curve present no polarization. The GCD tests were operated under various current densities ranging from 2 mA/cm^2^ to 32 mA/cm^2^ (Figure 4a) and the corresponding specific capacitance are listed in Figure 4b, expressing that high capacitance of 302.4 mF/cm^2^ was achieved under 2 mA/cm^2^. Under the high current density of 32 mA/cm^2^, the device still maintained 54.30% of the capacitance (164.2 mF/cm^2^), confirming its good rate capability. The cycling performance was also investigated by running charge/discharge cycling at a current density of 8 mA/cm^2^ using GCD method (Figure 4c). After 5000 cycles, the device still kept 91% of the origin capacitance, demonstrating its excellent cycling performance.

In order to evaluate the feasibility of our constructed N/Ar-6//AC-40 asymmetric device, the power density and energy density calculated through Equations (6) and (7) were compared with other Co_3_O_4_-based supercapacitive devices in previous reports. As displayed in Figure 4d, our device realized a high energy density of 80.5 Wh/kg at the power density of 1.3 kW/kg, while the power density reached 25.4 kW/kg at the energy density of 43.7 Wh/kg. The comprehensive performance is superior or comparable to the recently reported Co_3_O_4_//carbon devices, such as Co_3_O_4_ nanosheet (38.8 Wh/kg at 7.5 kW/kg) [41], Co-Co_3_O_4_ (60.8 Wh/kg at 5.8 kW/kg) [42], Co_3_O_4_ nanorod (34.1Wh/kg at 4 kW/kg) [43], Co_3_O_4_@C nanofiber (37.75 Wh/kg at 1.8 kW/kg) [44], Cl-doped Co_3_O_4_ nanosphere (74 Wh/kg at 0.807 kW/kg, 47 Wh/kg at 24.2 kW/kg) [45], Co_3_O_4_ nanoplate (66.1 Wh/kg at 1.652 kW/kg) [46], and Co_3_O_4_ nanosheet (51.7 Wh/kg at 1.125 kW/kg, 34.7 Wh/kg at 33.75 kW/kg) [47].

### 3.4. HER Performance

**Figure 5:**
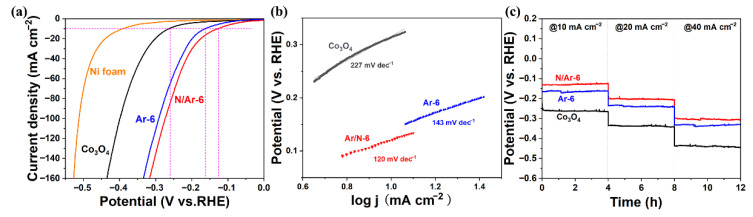
HER performance of Co_3_O_4_, Ar-6 and N/Ar-6: (**a**) LSV curves, (**b**) Tafel plots and (**c**) Chronopotentiometric curves at different current densities.

The electrocatalytic capability of the N/Ar-6 electrode toward HER was assessed by linear sweep voltammetry (LSV), whereas for comparison, pure Co_3_O_4_ and Ar-6 electrodes were also measured under the same conditions. As presented in Figure 5a, bare Ni foam showed unsatisfactory performance, while the hydrothermally growth of Co_3_O_4_ nanowires improved the performance since Co_3_O_4_ is a catalytic active material for HER electrocatalysis. Notably, the plasma treatment further boosted the performance. Especially for the N/Ar-6 electrode, the lowest overpotential (at the current density of 10 mA/cm^2^) of about 126 mV (vs. RHE) was obtained, which compares favorably to pure Co_3_O_4_ (263 mV vs. RHE) and Ar-6 (165 mV vs. RHE). To further perceive the reaction kinetics of the electrodes, Tafel plots (Figure 5b) were constructed from LSV curves. The N/Ar-6 electrode possesses a Tafel slope of 120 mV/dec, remarkably lower than that of pristine Co_3_O_4_ (227 mV/dec) and Ar-6 (143 mV/dec), elucidating the favorable kinetics for HER on the N/Ar-6 electrode. The electrochemical stability, which is an important criterion in evaluating the HER performance of the catalysts in practical applications, was studied using chronopotentiometry at constant current densities of 10, 20 and 30 mA/cm^2^. As shown in Figure 5c, the N/Ar-6 electrode keeps the steady potentials of 126 mV, 201 mV and 303 mV at the current density of 10, 20 and 30 mA/cm^2^, respectively, which is apparently lower than the potential of Co_3_O_4_ and Ar-6 at the same current density.

### 3.5. Mechanisam Study

According to the previous results, the NH_3_/Ar plasma treatment mainly works on Co_3_O_4_ nanowires in three aspects: (1) producing a porous surface and increasing the active surface area; (2) generating oxygen vacancies on the surface layer; (3) injecting nitrogen dopant into Co_3_O_4_ lattice. Since the first two aspects mainly originate from the etching effect of plasma and can be accomplished in a pure Ar atmosphere, the Ar-6 electrode was applied as the control group in exploring the relationship between structure and performance.

**Figure 6:**
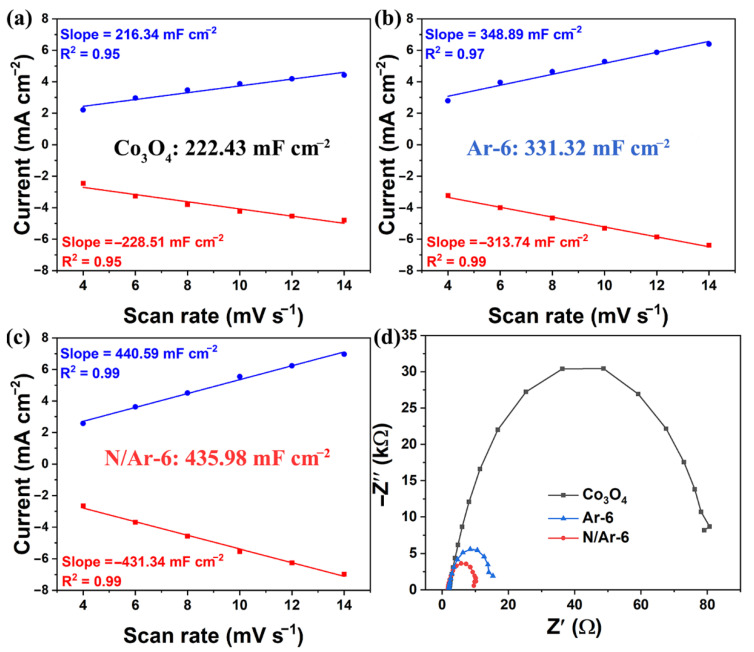
The calibration plots of anode and cathode currents (derived from Figure S7) vs. square root of scan rates: (**a**) Co_3_O_4_, (**b**) Ar-6 and (**c**) N/Ar-6. (**d**) Nyquist curves of Co_3_O_4_, Ar-6 and N/Ar-6.

First, the electrochemical active surface areas (EASA) of Co_3_O_4_, Ar-6 and N/Ar-6 electrodes were studied to verify the influence of etching and nitrogen doping. The CV curves of all electrodes were recorded in the non-faradic regions (0.05 V–0.15 V) under different scan rates (4–14 mV/s), as depicted in Figure S7a–c. By taking the anode and cathode current densities at 0.1 V, the slope was obtained after linear fitting (Figure 6a–c). The double-layer capacitance as a reference of EASA validates that the Ar-6 electrode (slope = 331.32 mF/cm^2^) possesses a higher EASA than Co_3_O_4_ (slope = 222.43 mF/cm^2^), indicating that the etching effect of plasma produced a higher surface area and abundant active sites. The highest slope (435.98 mF/cm^2^) achieved by the N/Ar-6 electrode corroborates the important role of nitrogen doping in activating reaction sites.

Furthermore, the EIS measurement was performed in a three-electrode system to study the electron/ion transfer behavior. The Nyquist plots in the high-frequency range are illustrated in Figure 6d. The value of equivalent series resistance (Rs) can be obtained from the X-intercept of the EIS curve. According to the Nyquist curves in Figure 6d, the value of Rs for Co_3_O_4_, Ar-6 and N/Ar-6 was calculated to be 2.16, 1.94 and 1.73 Ω, respectively. The slight decrease indicates that Rs is not sensitive to the plasma treatment since it is mainly composed of bulk phase resistance (the intrinsic resistance, electrolyte ionic resistance) and interfacial resistance between active materials and current collector, which is well protected from plasma. However, the charge-transfer resistance (R_ct_) was greatly affected by the plasma treatment, as demonstrated by the distinct size of the semicircles. The R_ct_ for Co_3_O_4_, Ar-6 and N/Ar-6 was determined to be 38.96, 5.90 and 3.64 Ω, respectively. The sharp drop of R_ct_ for both N/Ar-6 and Ar-6 implies that the superior electron transfer kinetics is mainly induced by oxygen vacancies. A further slight decrease in R_ct_ for N/Ar-6 indicates that the nitrogen incorporation into the Co_3_O_4_ lattice also facilitates the electron transfer.

## 4. Conclusions

In summary, Co_3_O_4_ nanowires were assembled on Ni foam by the hydrothermal method and surface-modified by NH_3_/Ar plasma treatment. Comparative analyses were conducted on pristine Co_3_O_4_, Ar-6 and N/Ar-6. N/Ar-6 outperformed all the other samples and exhibited the superior supercapacitive and electrocatalytic performance, which should be accredited to the synergistic effects of plasma etching and nitrogen doping, offering copious active reaction sites and favoring the electron transfer kinetics. Our low-temperature modification method and relevant investigation of the synergistic effect of etching and nitrogen doping provides the necessary experimental and theoretical foundation for the future design of convenient, low-cost and high-performance electrochemical devices.

## Data Availability

Not applicable.

## Supplementary Materials

The following supporting information can be downloaded at https://www.mdpi.com/article/10.3390/nano12172984/s1, Figure S1: SEM images of N/Ar-t (t = 2, 4, 8, 10 min); Figure S2: EDS mapping of N/Ar-t (t = 2, 4, 6, 8, 10 min); Figure S3: XPS survey spectra of Co_3_O_4_, Ar-6 and N/Ar-6; Figure S4: (a) CV and (b) GCD curves of pristine Co_3_O_4_ and N/Ar-t (t = 2, 4, 6, 8, 10 min); Figure S5: (a) CV, (b) GCD and (c) Nyquist curves of AC-40 half cell; Figure S6: CV curves of N/Ar-6//AC-40 asymmetric device; Figure S7: CV curves of Co_3_O_4_, Ar-6 and N/Ar-6 for EASA measurement. Table S1: Element concentration in N/Ar-t (t = 2, 4, 6, 8, 10 min).
