# Supplementary material for: Plasma-Engineered N-CoOx Nanowire Array as a Bifunctional Electrode for Supercapacitor and Electrocatalysis
Source: Nanomaterials (Basel). 2022 Aug 29;12(17):2984. doi: 10.3390/nano12172984 (PMC9457654; doi:10.3390/nano12172984)
Supplement: Supplementary file 1 [file nanomaterials-12-02984-s001.zip › nanomaterials-1885055-supplementary.pdf]

Supplementary Materials

Plasma-engineered N-CoO_x nanowire array as bifunctional electrode for supercapacitor and electrocatalysis

Qi Wang, TongTong Zhong and Zhou Wang *

Key Laboratory of Liquid-Solid Structural Evolution and Processing of Materials of Ministry of Education, School of Materials Science and Engineering, Shandong University, Jinan 250061, China
* Correspondence: wangzhou@sdu.edu.cn

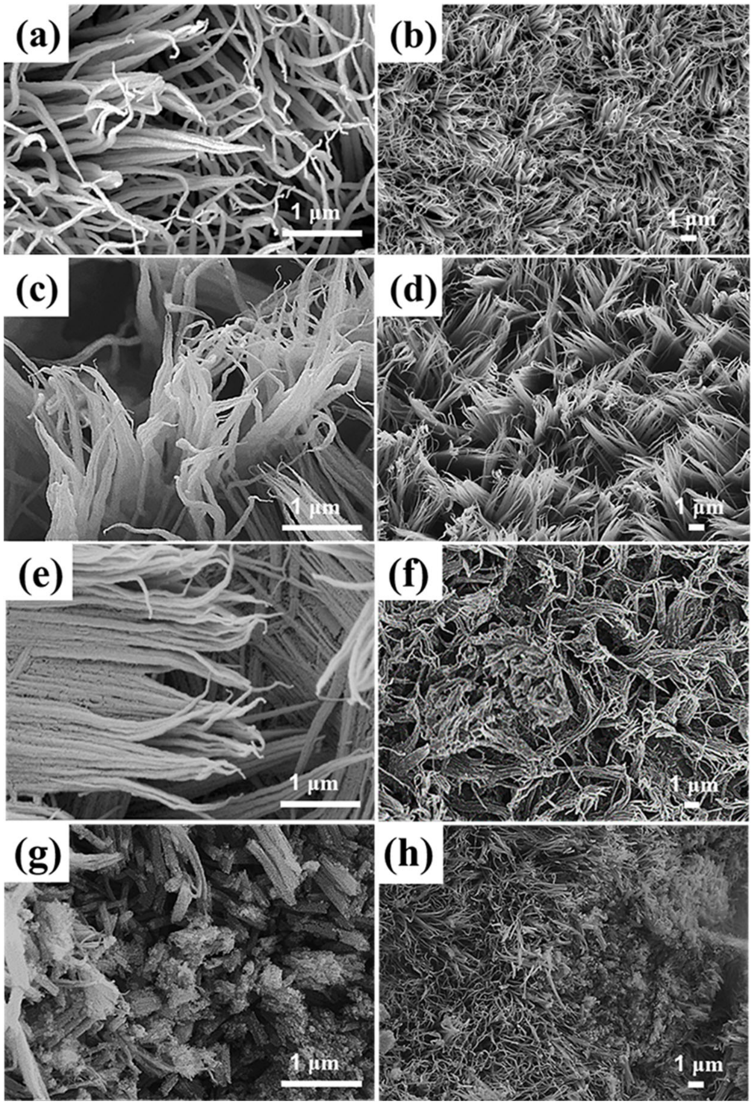

Figure S1. SEM images of N/Ar-t: (a,b) t = 2, (c,d) t = 4, (e,f) t = 8, (g,h) t = 10.

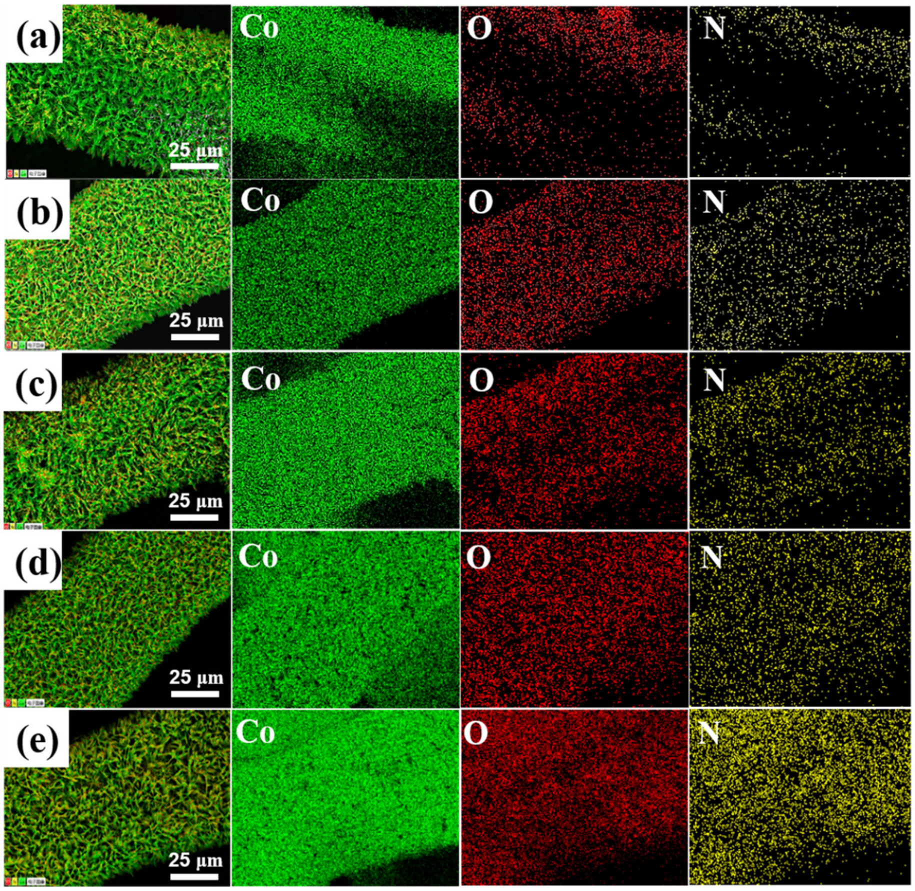

Figure S2. EDS mapping of N/Ar-t: (a) t = 2, (b) t = 4, (c) t = 6, (d) t = 8, (e) t = 10.

Table S1. Element concentration in N/Ar-t (t = 2, 4, 6, 8, 10 min)

Sample	Co (wt%)	Co (at%)	O (wt%)	O (at%)	N (wt%)	N (at%)
N/Ar-2	99.4	97.7	0.6	0.3	0	0
N/Ar-4	98	92.9	1.7	6.0	0.3	1.1
N/Ar-6	98.5	94.8	1.1	3.9	0.3	1.3
N/Ar-8	98.8	95.1	0.9	3.2	0.4	1.7
N/Ar-10	97.8	92.4	1.6	5.6	0.5	2

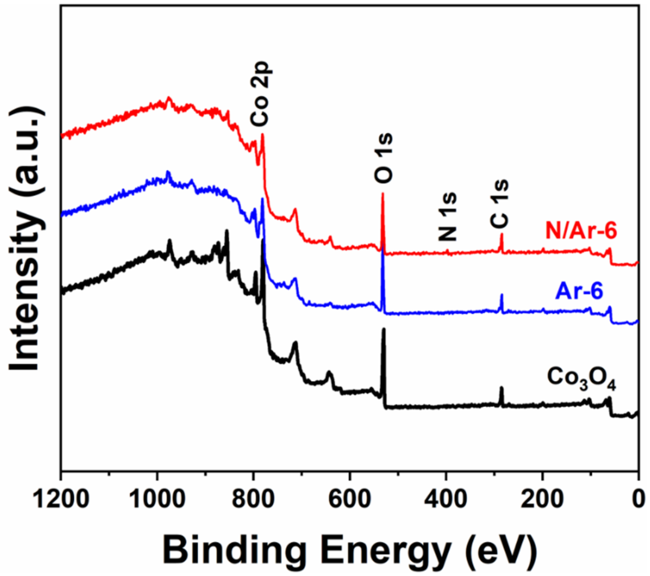

Figure S3. XPS survey spectra of Co_3O_4, Ar-6 and N/Ar-6.

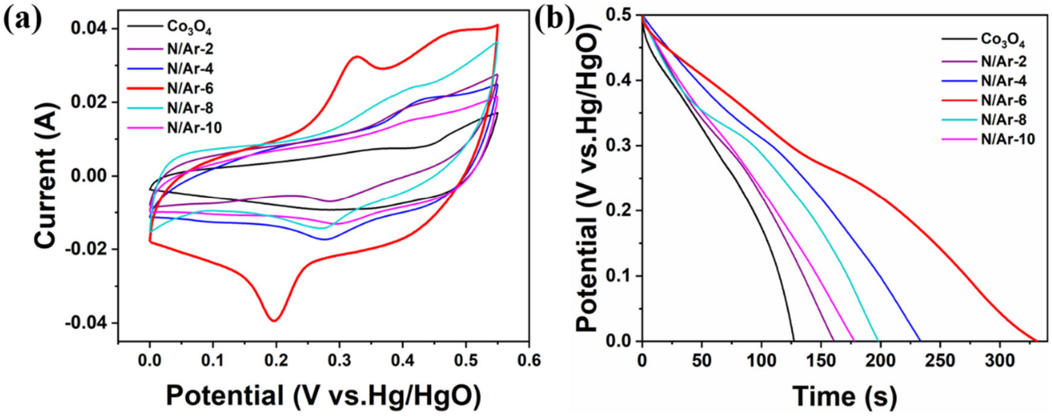

Figure S4. (a) CV and (b) GCD curves of pristine Co_3O_4 and N/Ar-t (t = 2, 4, 6, 8, 10 min).

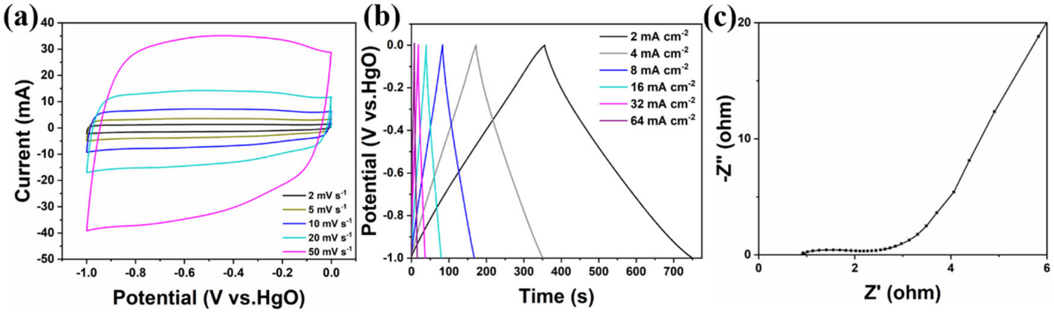

Figure S5. (a) CV, (b) GCD and (c) Nyquist curves of AC-40 half cell.

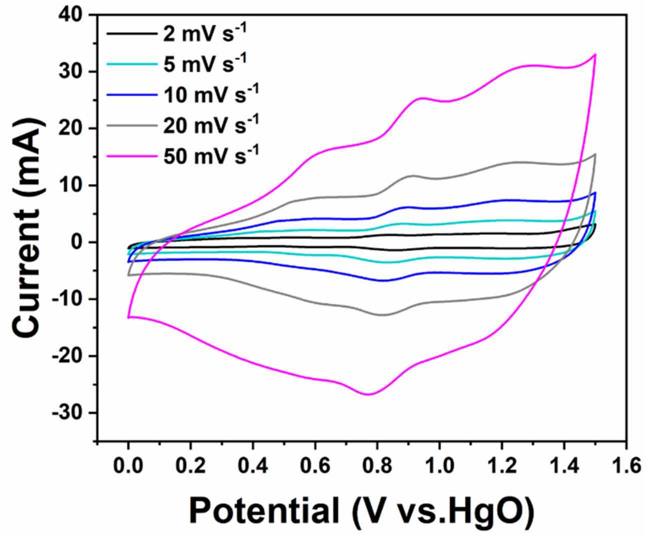

Figure S6. CV curves of N/Ar-6//AC-40 asymmetric device.

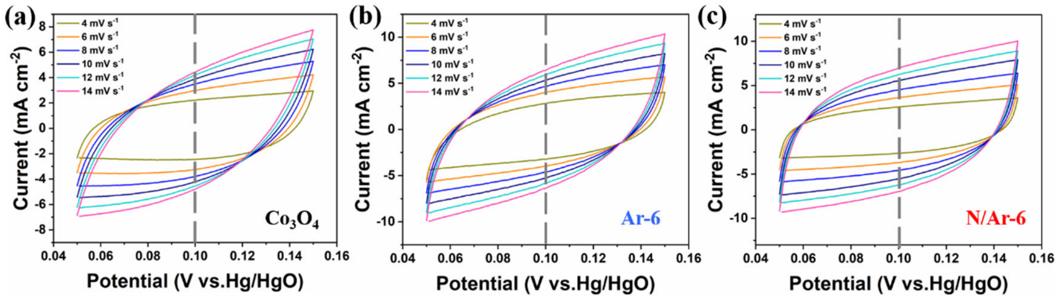

Figure S7. CV curves of Co_3O_4, Ar-6 an N/Ar-6 for EASA measurement.
